# Supplementary material for: A qualitative study of Chinese teacher’s perceptions and practices of meritocracy
Source: PLoS One. 2025 Apr 16;20(4):e0321424. doi: 10.1371/journal.pone.0321424 (PMC12002450; doi:10.1371/journal.pone.0321424)
Supplement: S1 Appendix — (DOCX) [file pone.0321424.s001.docx]

Appendix: **Interview questions.**

| **Topic** | **Questions** |
| --- | --- |
| Warm up  暖场 | In your opinion, what leads to educational success and why?  在学校中，哪些因素对学生学业成功影响最大？为什么？ |
|  | What is your view of the saying“hard work guarantees reward?”  你如何看待“天道酬勤”这句话？ |
| Responses to meritocracy in school  **关于学校中优绩主义的回应** | Based on your observations, what might explain the underperformance of certain students in your class?  在你的班级中，如果一个学生学习成绩很差，你觉得原因是什么？ |
|  | What advantages and barriers might a high-achieving student from a working-class family encounter?  如果一个学生出身普通但成绩优秀，你觉得他可能有哪些优势又会遇到那些阻碍？ |
|  | The phenomenon“high-achieving students from underprivileged backgrounds”has gained public attention. What is your view on the phenomenon?  你知道“寒门贵子”这种说法吗？你如何看待这种现象？ |
| Responses to nonmeritocratic factors  **关于非优绩性因素的回应** | What contributes to people’s achievement of prestigious occupations and why?  你认为哪些因素会影响有声望职位的获得？为什么？ |
|  | To what extent does the claim that “education serves as an engine of social mobility” align with empirical reality?  你如何看待“教育改变命运”这句话？它与现实相符吗？ |
|  | What advantages and barriers might a graduate of a prestigious university from a modest family encounter?  如果一个名校毕业但家境普通的学生，你觉得在进入职场后会有哪些优势和阻碍？ |
| **Teaching practices**  **教学实践** | Have you experienced tension between merit-based evaluation and compensatory interventions for disadvantaged groups? If so, how were these tensions reconciled?  在坚持优绩主义还是考虑非优绩主义因素中，您是否遇到过挑战？您是如何应对的？ |
|  | What are your interventions for students who are affected by uncontrollable factors such as family environment and economic conditions? Can you please provide an example?  对于那些受家庭环境、经济条件等非能力因素影响的学生，您会采取哪些干预方式？您能举一个例子吗？ |
