# Supplementary material for: A qualitative study of Chinese teacher’s perceptions and practices of meritocracy
Source: PLoS One. 2025 Apr 16;20(4):e0321424. doi: 10.1371/journal.pone.0321424 (PMC12002450; doi:10.1371/journal.pone.0321424)
Supplement: Attachment — Submitted filename: Response to Reviewers(minor revised).docx [file pone.0321424.s006.docx]

**Response to Reviewer comments**

Dear Reviewer:
Thank you for your comments concerning my manuscript entitled “When Meritocratic Beliefs Collide: A Qualitative Study of Chinese Teachers’ Dilemmas (ID: PONE-D-24-28202)”. Those comments are all valuable and very helpful for revising and improving the paper. We have studied the comments carefully and have made a revision which we hope to meet with approval. Revised portions are highlighted in yellow on the paper. The main corrections in the paper and the responses to the reviewer’s comments are as flowing.

| **Reviewer ’s comments** | **Authors response** |
| --- | --- |
| **(#1) Methods.** The “Method - Participants” section fails to provide any information on which to compare personal characteristic distributions of the sampled teachers in each of the 3 schools. | Regarding the suggestion, we provided basic information about the participating schools and indicated the personal characteristics of the sample teachers in Table 1. |
| Why are the interview questions only broadly described in“Method - Interview Outline”, rather than provided in the text or as an appendix? | As recommended, we have provided a complete of interview questions in Appendix. |
| It is unclear whether the interviews were conducted separately by the researchers or whether all were present for each. If conducted separately, please describe any procedures that were undertaken to ensure or check comparability between data collected by different interviewers. | Based on the suggestions, we have added more details of interview implementation. In general, consistency was enhanced through three measures: brief pre-interview training meeting, sharing of audio recordings, and mid-interview discussions to adjust methods. |
| it is unclear whether all data were coded independently by all researchers (either the 3 interviewers or the 2 authors listed on the paper or some other group). A consensus coding process for arriving at final codes is described, but not in enough detail to fully appreciate. | Thank you for your suggestions. We have detailed the analysis procedures, including measures to assess intercoder reliability and the validation of the coding framework. |
| **Results.** it appears 150 RMB should be referred to as the poverty line for per capita *daily* income. | According to official information, 150 RMB refers to monthly income |
| Is the sentence “students attribute academic success to three parts talent and seven parts effort.” a common proverb in China or is that based on some data from this or another study? It is a compelling way to describe the situation, but lacks justification as a categorical statement unless some context or citation is added. | This sentence comes from the respondent’s experienced-based statement. We have added context to it for clarity. |
| Please provide more context or description related to “ren qing shi gu” for readers not familiar with Chinese language and culture. | We have outlined *renqing*'s conceptual evolution from empire China to communist China, as well as its connection to *rguanxis* in the contemporary. |
| **Discussion.** It would be helpful there to either explicitly state that similar patterns of responses were observed across schools or (alternatively, if they were not similar) to explicitly highlight school differences. | Thank you for this insightful suggestion. Exploring potential causal relationships between teacher attitudinal variations and school types is indeed a compelling direction for future research. In the current study, however, we have adopted a restrained stand to discuss school differences due to the limited sample size of 24 teacher interviews. A broader samples in the future work may yield more solid evidence to state the connections between responses patterns and school types. |
| it would benefit from a systematic discussion (ideally with a conceptual diagram) of how different aspects of advantage/privilege (pre-education and educational home resources vs. socialization vs. guanxi/social networks) and merit (talent vs. effort) are seen as contributing to student’s educational achievement, as well as a clear comparison between that and the contributions to occupational success. | We have added a conceptual diagram illustrating teachers how teachers view the relationship between family background, *guanxi*, and academic/career success. The diagram clearly shows the contradictions in teachers’ perceptions regarding merit-based versus non-merit pathways in educational/career attainment, with comparative analysis of family background, ability, and *guanxi* shape outcomes through different ways in various contexts. |
| **(#2) Discussion.** Clarify the causal relationship between student background advantages and effort in determining success. | We clarified the relationship between family background advantages and effort in educational success. In China, family background can affects students’ academic performance. However, the exam-oriented education system limits the benefits of family background advantaged (Byun et al, 2012) ,making effort equally important for both advantaged and disadvantaged students.  Byun, S. Y., Schofer, E., & Kim, K. K. (2012). Revisiting the role of cultural capital in East Asian educational systems: The case of South Korea. Sociology of education, 85(3), 219-239. |
| Highlight how teachers emphasize effort as the key factor influencing student outcomes, even though structural constraints exist. | Based on the teachers’ responses, we have emphasized the paradox of rewarding effort while prioritizing its moral value over practical outcomes, demonstrating how they navigate meritocracy in teaching practices. |
| Expand the discussion on structural constraints and their impact on teachers’ agency | We have interpreted dual constraints: macro-level national policies ( control and obedience principle educational governance) that constrain teacher autonomy, and micro-level institutional assessment (performance metrics) intensified workload. |
| Provide a more in-depth exploration of the implications for policy and practice. | We have deliberately reduce policy recommendations given persistent structural issues in China, as key implications remain self-evident yet unaddressed. From a practical view, while this study may enable educators to reflect on their ingrained perceptions, such reflective capacity on the basis of both professional agency and relief from administrative overload. Thus, we keep our discussion of practical and policy implications brief. |
| Special thanks to you for your insightful comments. | |

**Reviewer:**

    We made some changes in the manuscript. These changes will not influence the content and framework of the paper. And here we did not list the changes but marked them in yellow in the revised paper. We have supplemented the data availability statement for this study, which is not included in this text but will be submitted as supplementary materials through the submission system. I appreciate for Editors/Reviewers’ warm work earnestly and hope that the correction will meet with approval.

Once again, thank you very much for your comments and suggestions.

Yours sincerely,
