# Supplementary material for: A qualitative study of Chinese teacher’s perceptions and practices of meritocracy
Source: PLoS One. 2025 Apr 16;20(4):e0321424. doi: 10.1371/journal.pone.0321424 (PMC12002450; doi:10.1371/journal.pone.0321424)
Supplement: Attachment — Submitted filename: Comments.docx [file pone.0321424.s002.docx]

Dear Editor,

Thank you for providing me with the opportunity to review this manuscript. I have read the complete manuscript and found it to be of good quality. However, there is always room for improvement. Below are some changes that need to be incorporated before publication:

- The abstract is very brief and not well-written. It should be expanded and improved for clarity.
- The introduction is overall well-written and logically coherent. However, it lacks the latest references, which could help strengthen the research gap.
- In the literature review, the authors developed strong arguments on "Meritocracy and Education," "Teachers’ Perceptions of and Practices Regarding Meritocracy," and "Meritocracy in the Chinese Context." However, there are no recent references that support the literature with relevant theories.
- The methods section should be clearer to the reader. On what basis were these school teachers from Shenzhen City selected? This section must be more understandable.
- The results section is well-written and well-explained by the authors. The proposed questions are clearly addressed and interpreted effectively.
- Limitations and future directions are missing and should be included.

Thanks

Muhammad Naseem Abid
