# Supplementary material for: A qualitative study of Chinese teacher’s perceptions and practices of meritocracy
Source: PLoS One. 2025 Apr 16;20(4):e0321424. doi: 10.1371/journal.pone.0321424 (PMC12002450; doi:10.1371/journal.pone.0321424)
Supplement: Attachment — Submitted filename: Response to Reviewers.docx [file pone.0321424.s005.docx]

**Response to Reviewer comments**

Dear Reviewer:
Thank you for your comments concerning my manuscript entitled “A Qualitative Study about Chinese Teacher’s Perceptions and Practice of Meritocracy(ID: PONE-D-24-28202)”. Those comments are all valuable and very helpful for revising and improving the paper. We have studied the comments carefully and have made a revision which we hope to meet with approval. Revised portions are highlighted in yellow on the paper. The main corrections in the paper and the responses to the reviewer’s comments are as flowing.

**Publishing Editor**

| A rebuttal letter that responds to each point raised by the academic editor and reviewer(s). A marked-up copy of your manuscript that highlights changes made to the original version. | We carefully considered all reviewer comments when we revised the whole manuscript. We will submit a list of responses along with revised manuscript. |
| --- | --- |

**Reviewer:**

| **Reviewer’s comments** | **Authors response** |
| --- | --- |
| **Abstract.** The authors need to focus on improving the abstract. As the first part of the manuscript, it is crucial for capturing the reader's attention. In my opinion, the abstract is too brief. Please consider making it more engaging and comprehensive. | As the reviewers suggested that we have rewrite for its clarity. |
| **Introduction.** The introduction is overall well-written and logically coherent. However, it lacks the latest references, which could help strengthen the research gap. | Regarding the suggestion about the introduction, we introduced new literature on Chinese attitudes toward meritocracy. We analyzed the emerging ‘lying flat’ movement, the varying findings on people’s meritocratic beliefs in unequal environments, and changes in the labor market. |
| **Literature review.** The authors developed strong arguments on "Meritocracy and Education," "Teachers’ Perceptions of and Practices Regarding Meritocracy," and "Meritocracy in the Chinese Context." However, there are no recent references that support the literature with relevant theories. | The discussion of meritocracy has produced many classic theories, meaning that some dated studies are inevitably referenced. However, we have still include the latest research to provide fresh insights. Such as, ideas about meritocracy from Platz (2020, 2022) and Mulligan (2018).  For the part of “Meritocracy and Education”, we included ideas about meritocracy as a society system from Bandiera (2024), Moreira (2022), Liu(2023) and Bruni (2021).  We added ideas about school meritocracy and teachers’ perceptions and practices from recent references,such as Castillo et al. (2022), Gonçalves et al.(2024), Krumer (2022) and Doyle et al. (2023).  Regarding “Meritocracy in the Chinese Context,” we have added the typical features of meritocracy in China. We would like to clarify that we have included recent research and official data to support the arguments, such as the employment of college graduates in 2022 and the number of graduate students enrolled in 2023. |
| The concept of meritocracy is not adequately explained. Since it is the central theme of the manuscript, the authors must provide a more detailed and thorough explanation of this concept. | Regarding the suggestion about the literature, we restructured this section. We have thoroughly explained the concept of meritocracy from both the philosophical perspective of justice theory and as a social model. Using the theory of desert, we have clarified what merit means in the context of distributive justice. As a social model, we discussed the potential political and economic effects of a society sorted around merit. |
| Why are meritocracy and education important? What are the reasons behind this? The authors should focus on these questions and support their discussion with relevant and recent literature, as many studies have explored meritocracy. | We utilized Bell’s classic theory, education-based meritocracy, to discuss the role of education in meritocracy. Our discussion is also combined with the arguments of lately studies to support the view of this paper that education is the cornerstone of meritocracy. |
| Why focus only on teachers' perceptions and practices regarding meritocracy? The rationale behind this focus must be clarified, using the latest and relevant references. | We have rephrased this section to explain why we focus on teachers. After briefly outlining the foundational role of schools in promoting meritocracy, we then analyzed the influence of teachers’ behaviors and views in this process.  Example:  Schools’ contribution to meritocracy is closely linked to teachers (Castillo et al, 2022; Gonçalves et al., 2024), making their practices and beliefs key to understanding meritocracy and social inequity.  An international study by Batruch et al. (2023) found that teachers with meritocratic beliefs, rather than perceiving unfairness and supportive of affirmative action policies, encourage students to work hard, thereby perpetuating and strengthening meritocratic beliefs within schools. |
| The concept of meritocracy in the Chinese context must be clearly articulated and supported with appropriate references. | Based on existing research, we summarized the characteristics of meritocracy in China, highlighting its features as a governance approach. |
| **Methods.** The methods section is insufficient and requires a more detailed explanation. | Regarding the suggestion on how to invite participants, we have added a brief description outlining the criteria followed for inviting participants in our study.  We have detailed the coding process and identified themes on the basis of the concept of meritocracy and teachers’ perceptions and teaching practices. The data were thematically coded according to meritocratic and non-meritocratic factors within the concept of meritocracy. The secondary coding refined the following themes: views of meritocratic factors and Guanxi, and teaching practices, considering that Guanxi were frequently mentioned. |
| **Discussion.** The limitations of the study also need to be discussed in this section. | We have added a new paragraph to discuss the limitations of the study. |
| Special thanks to you for your good comments. | |

    We made some changes in the manuscript. These changes will not influence the content and framework of the paper. And here we did not list the changes but marked them in yellow in the revised paper. I appreciate for Editors/Reviewers’ warm work earnestly and hope that the correction will meet with approval.

Once again, thank you very much for your comments and suggestions.

Yours sincerely,
