# Supplementary material for: A qualitative study of Chinese teacher’s perceptions and practices of meritocracy
Source: PLoS One. 2025 Apr 16;20(4):e0321424. doi: 10.1371/journal.pone.0321424 (PMC12002450; doi:10.1371/journal.pone.0321424)
Supplement: Attachment — Submitted filename: Review Comments - PONE-D-24-28202R1.docx [file pone.0321424.s004.docx]

Comments to the author are presented based on the PLOS reviewer criteria below:

1. The study presents the results of original research.

Yes, the study presents results based on a new set of interviews collected for the purposes of the study.

2. Results reported have not been published elsewhere.

Correct.

3. Experiments, statistics, and other analyses are performed to a high technical standard and are described in sufficient detail.

This criterion is the least clearly met, based on my expertise and reading (as a methodologist). The following concerns should be addressed prior to publication:

a. The “Method - Participants” section is improved but fails to provide any information on which to compare personal characteristic distributions of the sampled teachers in each of the 3 schools. If comparisons are to be made on the basis of school, then some table of basic descriptives (aggregated to the level of school, rather than individual, to preserve privacy) would help evaluate whether purported differences related to school could alternatively be explained by differences in gender, education, teaching experience and position. In the discussion, however, little attention seems to be paid to which school (other than to report). It would be helpful there to either explicitly state that similar patterns of responses were observed across schools or (alternatively, if they were not similar) to explicitly highlight school differences the way you highlight differences in how teachers talk about high-performing and low-performing students.

b. Why are the interview questions only broadly described in “Method - Interview Outline”, rather than provided in the text or as an appendix (ideally in both Mandarin and English, given that you are seeking to publish results in an English-language journal)? Subtle variations in question wording can elicit significantly different responses. (It is not necessary to include the questions used in the pilot interviews, as that data was only used in developing the final interview questions).

c. In “Method - Procedure”, it is unclear whether the interviews were conducted separately by the “three experienced qualitative researchers” or whether all were present for each. If conducted separately, please describe any procedures that were undertaken to ensure or check comparability between data collected by different interviewers.

d. In “Method - Analysis”, it is unclear whether all data were coded independently by all researchers (either the 3 interviewers or the 2 authors listed on the paper or some other group). A consensus coding process for arriving at final codes is described, but not in enough detail to fully appreciate.

4. Conclusions are presented in an appropriate fashion and are supported by the data.

Overall, the paper is well-presented aside from the comments elsewhere in this review. However, it would benefit from a systematic discussion (ideally with a conceptual diagram) of how different aspects of advantage/privilege (pre-education and educational home resources vs. socialization vs. guanxi/social networks) and merit (talent vs. effort) are seen as contributing to student’s educational achievement, as well as a clear comparison between that and the contributions to occupational success. This might help resolve some tensions in the text, for example the causal ordering between student personal/home advantage and effort in leading to success. In one model, students that have antecedent conditions for success then can distinguish themselves and actually succeed based on effort. Alternatively, all students have the ability to work hard and meet some degree of success in school, but the final impact of that success on their personal and career outcomes is limited by non-meritocratic factors. Both models integrate merit and non-merit factors, but they tell different stories about their roles. Such analysis or diagram may also help with one of the most compelling, if not fully explored, observations from the data - that teachers somewhat uniformly teach as if effort was the most important thing, on the assumption that it’s the primary factor they might influence in their students.

5. The article is presented in an intelligible fashion and is written in standard English.

Some specific suggestions to improve the language:

a. On page 12, it appears 150 RMB should be referred to as the poverty line for per capita *daily* income (per capita net income is ambiguous and could refer to annual per capita income)

 b. Please provide more context or description related to “ren qing shi gu” for readers not familiar with Chinese language and culture if you are going to emphasize it on p. 15. “Worldly wisdom” is ambiguous in English and the Chinese concept (from what I see in a search online) encompasses multiple related meanings, so it’s important to understand how it is actually being used.

c. On page 13, there is an assertion that “students attribute academic success to three parts talent and seven parts effort.” Is that a common proverb in China or is that based on some data from this or another study? It is a compelling way to describe the situation, but lacks justification as a categorical statement unless some context or citation is added.

6. The research meets all applicable standards for the ethics of experimentation and research integrity.

Yes.

7. The article adheres to appropriate reporting guidelines and community standards for data availability

Reporting is adequate, other than the points above in section 3.

The standard PLOS Data Policy would require authors to share anonymized transcripts through an Open Science Framework Project or the Qualitative Data Repository or something similar, both for transparency and potential reuse. However, such sharing would only be possible if approved by the research ethics board and consented to by participants. If that is not possible, at a minimum, the descriptives table suggested in section 3a above would still provide some summary information. Additionally, a statement on data availability should be included, regardless of the level of availability.
